# THz-TDS Reflection Measurement of Coating Thicknesses at Non-Perpendicular Incidence: Experiment and Simulation

**DOI:** 10.3390/s21103473

**Published:** 2021-05-16

**Authors:** Ruben Burger, Julia Frisch, Matthias Hübner, Matthias Goldammer, Ole Peters, Enno Rönneberg, Datong Wu

**Affiliations:** 1 Department of Applied Sciences and Mechatronics, Hochschule München University of Applied Sciences, Lothstrasse 34, 80335 Munich, Germany; ruben.burger@hm.edu (R.B.); huebner1@hm.edu (M.H.); 2 Siemens Technology, Siemens AG, Otto-Hahn-Ring 6, 81739 München, Germany; julia.frisch@ph.tum.de (J.F.); matthias.goldammer@siemens.com (M.G.); 3 Chair of Non-Destructive Testing, Technical University of Munich, Franz-Langinger-Str. 10, 81245 Munich, Germany; 4 Menlo Systems GmbH, Bunsenstraße 5, 82152 Martinsried, Germany; o.peters@menlosystems.com (O.P.); e.roenneberg@menlosystems.com (E.R.)

**Keywords:** nondestructive evaluation, THz time-domain spectroscopy, layer thickness measurement, thermal barrier coatings, surface roughness, porosity, polarization, yttria-stabilized zirconia

## Abstract

Time-domain spectroscopy (TDS) in the terahertz (THz) frequency range is gaining in importance in nondestructive testing of dielectric materials. One application is the layer thickness measurement of a coating layer. To determine the thickness from the measurement data, the refractive index of the coating layer must be known in the surveyed frequency range. For perpendicular incidence of the radiation, methods exist to extract the refractive index from the measurement data themselves without prior knowledge. This paper extends these methods for non-perpendicular incidence, where the polarization of the radiation becomes important. Furthermore, modifications considering effects of surface roughness of the coating are introduced. The new methods are verified using measurement data of a sample of Inconel steel coated with yttria-stabilized zirconia (YSZ) and with COMSOL simulations of the measurement setup. To validate the thickness measurements, scanning electron microscopy (SEM) images of the layer structure are used. The results show good agreement with an average error of 1% for the simulation data and under 4% for the experimental data compared to reference measurements.

## 1. Introduction

Terahertz (THz) radiation bridges the gap between microwaves at the lower end and infrared radiation at the higher end with a frequency range between 0.1  THz and 30 THz [1]. The absorption of THz radiation by water vapor is strong, which leads to a reduced range in air [2]. While the penetration depth in conductors is negligible, THz radiation allows investigation of non-metal functional materials, including ceramics, semiconductors, fiber composites and polymers [1]. Many chemical compounds have spectroscopic fingerprints in the THz range, enabling remote detection methods [3]. Several medical applications are currently under investigation [4].

An important step for the use of THz in nondestructive testing (NDT) was the development of THz time-domain spectroscopy (THz-TDS), beginning in the 1980s, which is coupled to the development of frequency-stable, femtosecond-pulsed laser sources [5]. Today, a wide range of use cases [6] and techniques [7] have been demonstrated, including the development of THz-based computed tomography [8].

One application for THz-TDS in reflection mode is the non-contact, nondestructive measurement of coating thickness. Here, two approaches have been developed: a direct measurement in cases where the coating is thick enough to allow the separation of pulses [9,10] and model based methods that also allow measurement of thin, multi-layered coatings with optimization algorithms [11].

An important use case for a direct approach is the measurement of coatings for turbine blades. For increasing efficiency of gas turbine engines, the operating temperature is usually beyond $1000{}^{\circ }C$ [12]. At such high temperatures, even highly specialized alloys with internal cooling quickly reach their limits [13]. Here, additionally, the use of thermal barrier coatings (TBC) on the turbine blade surface has been established to reduce the thermal conductivity and to avoid the oxidation of the base materials [14]. The thickness of the TBC layer is in the range of several hundred microns and is an important parameter that has to be monitored during production and maintenance.

As a coating material, yttria-stabilized zirconia (YSZ - $Y_{2}O_{3+}{\mathrm{ZrO}}_{2}$) is often chosen. A metallic bond coat (BC) lies between the base metal material and the TBC layer for further corrosion protection and better ceramic growth [15]. There are two coating processes, which are mainly used in manufacturing: air plasma spray and electron beam physical vapor deposition. However, these processes are difficult to control properly, which can result in variations in coating thicknesses and properties such as porosity and thermal diffusivity.

Based on THz-TDS measurement signals, coating thicknesses can only be determined with known refractive indices of the coating layers. For perpendicular incidence of the THz pulse, the refractive index can be obtained by analyzing measurement data [9].

In this study, the analytical method was extended for general non-perpendicular THz radiation incidence cases, where the polarization of the THz pulse waves needs to be considered. Furthermore, the coating surface roughness can play a role in determination of coating thicknesses. The new method was verified with measurement data of a sample of YSZ TBC on Inconel steel and compared numerically with COMSOL simulations.

Following this introduction, the existing method is presented and then extended for the case of non-perpendicular incidence and non-negligible surface roughness in Section 2. The developed algorithm is then verified with simulated and real data in Section 3. A short summary and outlook concludes this work in Section 4. The Appendix A details the derivation of the presented modifications.

## 2. Materials and Methods

Fukuchi et al. [9] proposed a method to use spectral information of THz-TDS measurements to determine the refractive index of an examined sample coating from measurement data themselves. A short summary of the method is presented in this section followed by the expansion to a broader case in later sections.

### 2.1. Fukuchi Method at Perpendicular Incidence

**Figure 1:**
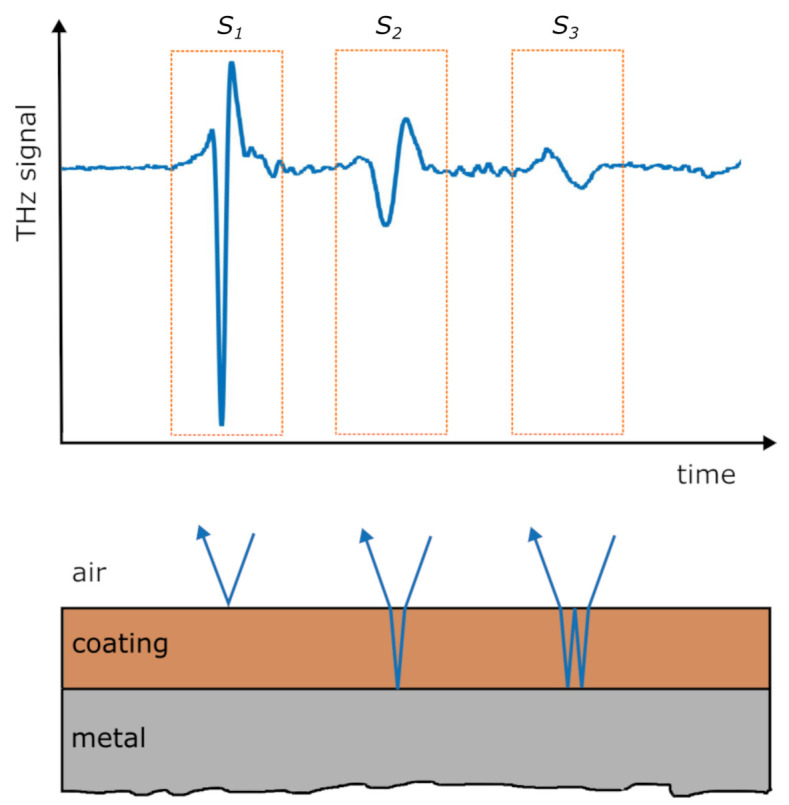
THz-TDS measurement data of sample “9 mils” (**top**) and constituting optical paths (**bottom**). In red: naming convention for the refractive index as well as reflection, transmission and absorption coefficients.

**Figure 2:**
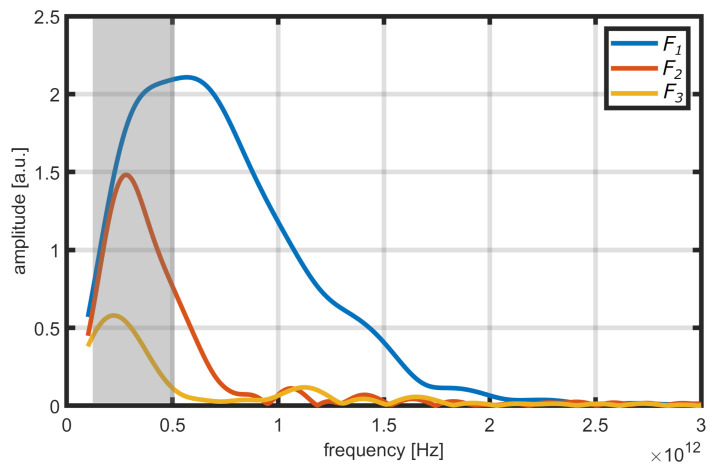
Frequency spectra $F_{i}$ of data from Figure 1 used in the method by Fukuchi et al. The frequency resolution was increased through zero-padding. The usable frequency range for the calculation of Γ is highlighted in grey.

The method proposed by Fukuchi et al. [9] is designed for THz-TDS measurements in reflection mode and at incidence of the THz radiation perpendicular to the sample surface. The surface of the sample is assumed as smooth and the layers as parallel. A typical THz-TDS signal of such a sample is shown in Figure 1. To calculate the refractive index, three pulses have to be extracted: the reflection at the air–TBC interface $S_{1}$, the reflection at the TBC–BC interface $S_{2}$ and the multiple reflection $S_{3}$ that twice traverses the TBC layer. The initial pulse $S_{0}$ does not have to be known. The Fourier transformations of the pulses $S_{i}$ are designated as $F_{i}$ and are shown in Figure 2. Through back-tracing of the optical path of the pulses, the frequency characteristics $F_{i}$ can be written as

(1) $$\begin{array}{cc} F_{1} & =-r_{ac}\;d_{a}^{2}\;F_{0}\\ F_{2} & =-t_{ac}\;r_{cm}\;t_{ca}\;d_{a}^{2}\;d_{c}^{2}\;F_{0}\\ F_{3} & =+t_{ac}\;r_{cm}^{2}\;r_{ca}\;t_{ca}\;d_{a}^{2}\;d_{c}^{4}\;F_{0} \end{array}$$

with $F_{0}$ as the frequency characteristics of the incident pulse and $r_{xx}$, $t_{xx}$ and $d_{x}$ as the (frequency dependent) reflection, transmission and absorption coefficients, respectively, of the material interfaces. The naming convention is shown in Figure 1. Through the combination of equations Equation (1) the incident pulse $F_{0}$ and the absorption coefficients $d_{x}$ can be eliminated

(2) $$\Gamma =\frac{F_{1}\;F_{3}}{F_{2}^{2}}=-\frac{r_{ac}\;r_{ca}}{t_{ac}\;t_{ca}}.$$

Γ is only dependent on the reflection and transmission parameters of the air-TBC interface. The Fresnel equations for perpendicular incidence connect the indices of refraction $n_{x}$ with $r_{x}$ and $d_{x}$ through

(3) $$\begin{array}{ccc} r_{ac}=\frac{n_{a}-n_{c}}{n_{a}+n_{c}}\; & & r_{ca}=\frac{n_{c}-n_{a}}{n_{c}+n_{a}}\\ t_{ac}=\frac{2\;n_{a}}{n_{a}+n_{c}}\; & & t_{ca}=\frac{2\;n_{c}}{n_{c}+n_{a}}. \end{array}$$

Insertion of Equation (3) into Equation (2) leads to

(4) $$\Gamma =\frac{{\left(n_{c}-n_{a}\right)}^{2}}{4\;n_{a}\;n_{c}}.$$

In most cases, $n_{a}=1$ can be assumed. (In environments of high humidity, the spectral refractive index of the air should be determined in a separate experiment.) Solving Equation (4) results in one physically meaningful solution

(5) $$n_{c}=1+2\;\Gamma +2\;\sqrt{\Gamma^{2}+\Gamma }.$$

Since Γ is calculated from measurement data, Equation (5) allows the calculation of the effective refractive index $n_{c}$ of the coating. The selection of a frequency range for the calculation of Γ has to be made carefully. The result will only be meaningful for frequencies with signal levels above noise for every reflection. Since reflection $F_{3}$ has the longest optical path, it will determine the frequency range. For the example in Figure 2, the limit will be around 0.5THz.

By measuring the time difference Δt between neighboring pulses, the thickness $h_{c}$ of the coating can be calculated through

(6) $$h_{c}=\frac{c_{vac}\;\Delta t}{2\;n_{c}}$$

with $c_{vac}$ being the speed of light in vacuum.

### 2.2. Adaptations for Inclined Incidence

THz-TDS measurements in reflection mode and perpendicular incidence have several drawbacks. The optical setup is often bulky and complicated. More importantly, the achievable beam power is reduced since a beam splitter is required in this configuration. An alternative is a measurement setup at angled incidence with physically separated excitation and detection of the THz pulse. To correctly use the method proposed by Fukuchi et al. [9] presented in the previous chapter for inclined incidence, several adaptations have to be made.

As additional parameters, the angle (relative to normal incidence) of the beam in air $\theta_{a}$ and in the coating $\theta_{c}$ appear in the general Fresnel equations. Both angles are connected through Snell’s law by

(7) $$n_{c}\;\sin\theta_{c}=n_{a}\;\sin\theta_{a}.$$

The experimental setup determines the angle $\theta_{a}$.

Furthermore, in the case of non-perpendicular incidence, the Fresnel equations differ depending on the polarization state of the incident beam. Thus, separate calculations have to be performed for parallel and perpendicular polarization and the result later averaged according to the polarization state of the incident beam. Since the equations are considerably more complicated to solve, the derivation of the formulas is relegated to Appendix A.1. The resulting index of refraction for perpendicular polarization is

(8) $$\begin{array}{cc} n_{⊥,c}= & n_{a}\sqrt{1+8\;\cos{\theta_{a}}^{2}\;\Gamma \left(\Gamma +1\right)+\cos{\theta_{a}}^{2}\;\sqrt{16\;\Gamma \;\left(4\;\Gamma^{3}+9\;\Gamma^{2}+4\;\Gamma +1\right)}}. \end{array}$$

For parallel polarization, an exact solution is not possible, but the following is a good approximation for sufficiently small $\frac{n_{a}}{n_{c}}$ and angles $\theta_{a}$ (compare Equation (A9))

(9) $$n_{\|,c}=\frac{n_{a}}{\cos\theta_{a}\left(2\;\Gamma +1-2\;\sqrt{\Gamma^{2}+\Gamma }\right)}.$$

**Figure 3:**
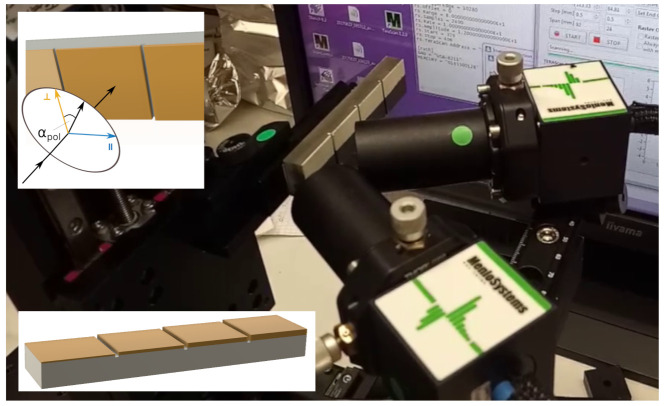
THz-TDS reflection mode setup with angled incidence. The sample is fixed on a scanning stage. The black coating on the bottom half is a graphite layer required for thermographic measurements (see [16]). Top inset shows the definition of the polarization components and of $\alpha_{pol}$. Bottom inset shows the sample geometry with scaled coating layer thicknesses for clarity.

For mixed polarization—that is, a linearly polarized incidence pulse with parallel and perpendicular polarization components—the refractive index can be calculated by superimposing calculations for $n_{⊥,c}$ and $n_{\|,c}$. For an angle of $\alpha_{pol}$ relative to perpendicular polarization (compare Figure 3), the effective refractive index $n_{\alpha_{pol}}$ is

(10) $$n_{\alpha_{pol}}=\frac{\sin\left(\alpha_{pol}\right)\;n_{\|,c}+\cos\left(\alpha_{pol}\right)\;n_{⊥,c}}{\left|\sin\left(\alpha_{pol}\right)\right|+\left|\cos\left(\alpha_{pol}\right)\right|}.$$

### 2.3. Time-of-Flight Correction

For perpendicular incidence, the thickness of the coating *d* can be calculated from the refractive index $n_{c}$ and the time between two consecutive reflections $\Delta t=t_{2}-t_{1}$ using

(11) $$d=\frac{1}{2}\;\frac{c_{vac}}{n_{c}}\;\Delta t.$$

**Figure 4:**
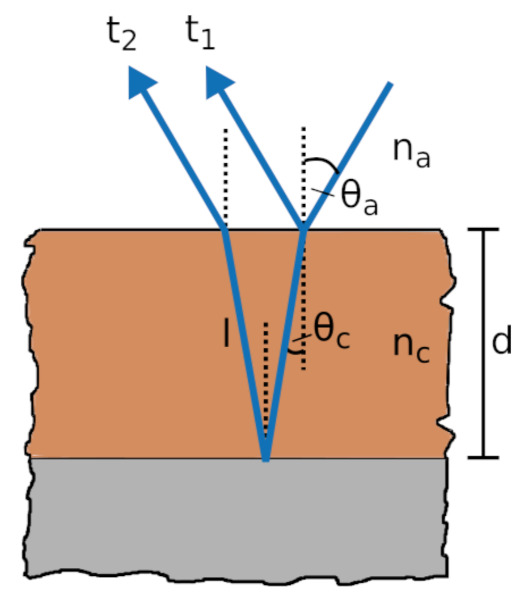
Path of THz pulse in coating at angled incidence.

The calculation has to be modified for the case of inclined incidence (see Figure 4). Here, Equation (11) gives the length of the optical path *l* in the material

(12) $$l=\frac{1}{2}\;\frac{c_{vac}}{n_{c}}\;\Delta t.$$

The optical path *l* is connected to the thickness through the angle in the coating $\theta_{c}$ by

(13) $$d=l\;\cos\theta_{c}.$$

Solving Equation (7) for $\theta_{c}$ and substituting the result together with Equation (12) into Equation (13) leads to a formula for the thickness *d* in case of inclined incidence

(14) $$d=\frac{1}{2}\;\frac{c_{vac}}{n_{c}}\;\Delta t\;\cos\left(\arcsin\left(\frac{n_{a}}{n_{c}}\;\sin\theta_{a}\right)\right).$$

In this work, the time difference Δt was determined with a modified impulse response algorithm [17], using the first reflection as the reference.

### 2.4. Surface Roughness Correction

Surface roughness affects the spectra of the reflected pulses [18,19]. The influence of the roughness for the determination of the refractive index of the coating was analyzed for the case of perpendicular incidence by Fukuchi et al. [20]. In this work, the roughness correction is calculated for inclined incidence using the Rayleigh roughness parameters of the interfaces. The derivation of the correction factor is based on previous works by Pinel et al. [21] and Piesiewicz et al. [22], which modeled the scattering and transmission of electromagnetic waves at rough interfaces. A detailed calculation of the correction factors can be found in Appendix A.4. Here, only the result is presented: the coefficient $\Gamma^{R}$, which is derived from the measured data, must be adjusted with the correction term $K_{R}$ to reconstruct the spectrum for smooth interfaces $\Gamma^{S}$ by

(15) $$\Gamma^{S}=\frac{1}{K_{R}}\;\Gamma^{R}$$

with

(16) $$K_{R}=\exp\left(-2\;{\left(k_{0}\;\sigma_{TBC}\right)}^{2}\left({\left(n_{a}\;\cos\theta_{a}\right)}^{2}-{\left(\frac{\left|n_{a}\;\cos\theta_{a}-n_{c}\;\cos\theta_{c}\right|}{2}\right)}^{2}+3{\left(n_{c}\;\cos\theta_{c}\right)}^{2}\right)\right)$$

and $\sigma_{TBC}$ being RMS roughness of the TBC interface.

The correction term $K_{R}$ is dependent on the roughness $\sigma_{TBC}$ of the TBC surface and on the refractive index $n_{c}$ of the TBC layer. Notably, the roughness of the TBC–BC interface is not present in $K_{R}$, because the contributions cancel each other out during the calculation. This is a very fortunate fact, since this surface is not accessible and the roughness is therefore not easily measurable. This leaves only the roughness of the top surface for measurement. This interface is accessible and can be inspected with commercial systems for roughness measurement. The roughness $\sigma_{TBC}$ has to be measured or estimated. The refractive index $n_{c}$, on the other hand, is the value that should be determined by the presented algorithm and is therefore unknown.

**Figure 5:**
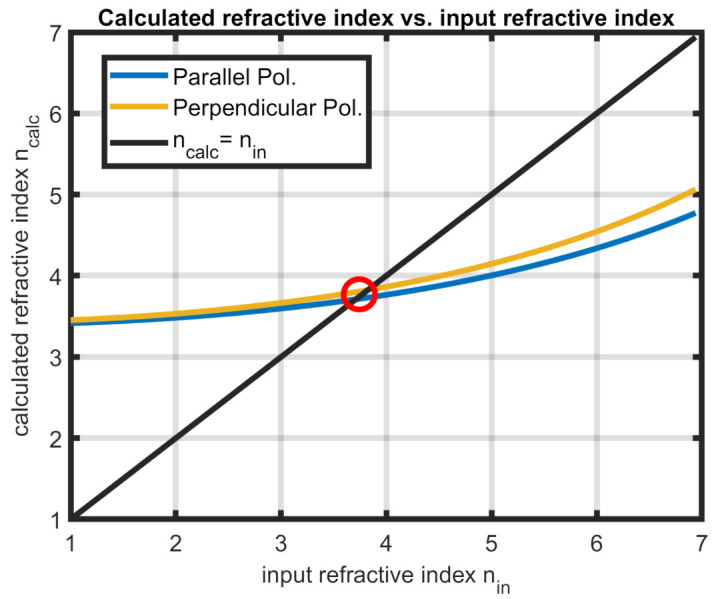
Iterative method for refractive index calculation. The intersection with the identity function gives a good estimate. The shown data are from a simulation with n=3.7.

However, through an iterative technique, a good approximation can be reached. For this, $K_{R}$ has to be calculated for a range of possible values of $n_{c}$. With this, the calculation for $n_{c}$ as presented in the previous sections is executed and averaged in the relevant frequency range. The key to finding the best estimate for $n_{c}$ is calculating the difference between the input and output refractive index, since, for the optimal value $n_{c,opt}$, the function must map to identity. By finding the point of minimal difference of input and output, the best approximation can be found (see Figure 5).

## 3. Results

In this section, the experimental THz-TDS setup and sample are presented, followed by a compact overview of the scanning electron microscopy (SEM) measurement and analysis. The COMSOL simulation of the THz-TDS measurement is introduced. The results of the application of the algorithms on simulation and on experimental data are compared with the reference values.

### 3.1. Experimental Setup and Used Sample

The investigated sample was an Inconel 738 substrate with metallic bond coat and YSZ layer manufactured with electron beam physical vapor deposition. The sample represents the layer structure of a turbine blade and was provided by Siemens Technology (Munich, Germany). It has been investigated in a previous study [16]. The sample comprises four steps (“6 mils”, “7 mils”, “9 mils” and “11 mils”) with different YSZ thicknesses ranging from nominal 6mil to 11mil or from 150μm to 280μm in SI units. (Mil $\equiv 10^{-3}\;$inch. The manufacturing parameters were specified in imperial units.) The exact manufacturing parameters are unknown. The sample is visible in Figure 3.

The experimental data in this study were gathered with the TERA ASOPS THz-TDS system manufactured by Menlo Systems (Martinsried, Germany) combined with two TERA15-FC antennas as emitter and receiver from the same manufacturer. This system uses the Asynchronous Optical Sampling (ASOPS) technique utilizing two mode-locked lasers emitting femtosecond pulses at λ=1560 nm with fixed repetition rate of 250 MHz and tunable phase difference. One laser is used for excitation, while the other is used for detection. This technique does not require mechanical delay stages. The laser pulses are delivered to the antennas via optical fiber. Generation and detection of THz pulses is based on the principle of the superconductive switch (Auston switch [5]). The system generates linearly polarized THz radiation with a bandwidth of 5 THz and a (THz) pulse energy of approx. 0.5 nJ. Lenses (TPX35) focus the pulses on the target surface with a diameter of approx. 1 mm at full bandwidth.

The THz-TDS system was set up for reflection mode measurement with angled incidence at approx. $30{}^{\circ }$ to the sample normal. The linearly polarized pulse had an angle relative to perpendicular polarization of approx. $\alpha_{pol}=20{}^{\circ }$. The setup is shown in Figure 3. The measurement was performed in a laboratory environment without evacuation or dry air/nitrogen purging.

**Figure 6:**
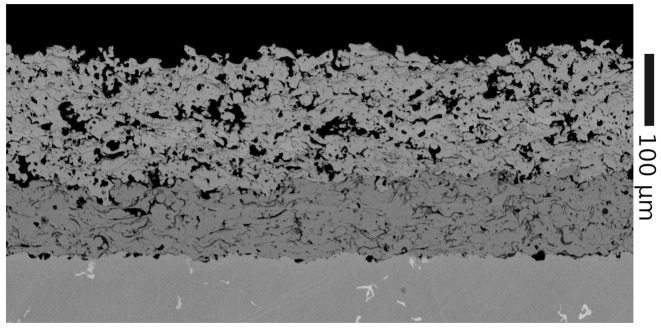
SEM images (@20kV) of sample “7 mils”. Layers (from top to bottom): resin (void), TBC, BC, Inconel steel base material.

Following the previous comparison of pulsed thermography and THz-TDS measurements on the sample by Frisch et al. [16] and within a second, forthcoming study by Frisch et al. [23], a cross-section cut of the sample was prepared and analyzed with SEM. The SEM measurement data were extracted from [23] and were used as the reference data for the validation of the presented adaption of the method proposed by Fukuchi et al. The SEM images were captured with the measurement software InTouchScope on the SEM JEOL JSM-6010 Plus (JEOL Ltd., Tokyo, Japan). Parameters were set at 20kV acceleration voltage, and high-vacuum and backscattered electron images were recorded. Figure 6 shows an example SEM scan.

### 3.2. SEM Image Analysis

The SEM images were analyzed and the thicknesses determined by visually averaging the air-TBC and TBC-BC interfaces and extracting the layer thickness with the SEM imaging software. Due to problems with charging of the sample during the SEM measurements, only one measurement could be performed for each of the four thickness steps. Since a statistical error cannot be determined, the uncertainty of the measurement was estimated as ±10μm.

The porosity analysis was performed with the image processing software ImageJ. To determine the porosity of the samples, the images were segmented using the modified IsoData-Algorithm [24]. For each sample, a polygon selection that covers a large part of the TBC area was traced. From this, the porosity was calculated as an area fraction. To determine the uncertainty of the measurement, for each SEM image, the porosity was determined in five arbitrary placed 150×150μm squares separately. The standard deviation of these values is used as the uncertainty of the porosity measurement. The results are collected in Table 1.

**Figure 7:**
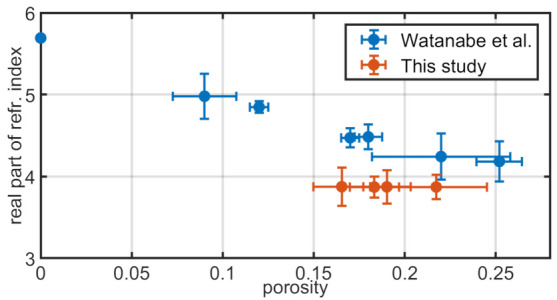
Comparison between calculated real parts of YSZ refractive index in relation to porosity. Values from Watanabe et al. [25] are measured at 0.5THz, while this study uses a ToF measurement.

Watanabe et al. [25] provide a thorough investigation of the dielectric properties of plasma-sprayed YSZ thermal barrier coating in the THz regime of 0.1–6.3 THz for a range of porosity in the microstructure. In the study, they found a high transmittance of frequencies around 0.5 THz, falling to almost zero at 1.5 THz. We can confirm this frequency range for our experimental data (see Figure 2). Watanabe et al. also provide measurements for the complex refractive index of YSZ layers in relation to the porosity ranging from bulk material (no porosity) to 25% porosity. A comparison between the real part of these results and the calculated effective refractive indices from our measurements is shown in Figure 7. The time-of-flight (ToF) calculation method used to determine the refractive index for the TDS data is presented in Appendix A.5. The uncertainty of the calculated refractive indices is determined via the propagation of uncertainty of Equation (A26) with errors for *h* (compare Table 1) and the error for the time difference between pulses Δt estimated as ±0.2 ps.

The values for the refractive index of YSZ measured in this study are 10–15% lower than those of [25] and do not reproduce the expected inverse relationship in relation to the porosity. A possible reason could be a higher statistical spread of the porosity measurement via SEM images. For the following simulations, the averaged refractive index of 3.87 was used.

The extraction of the surface roughness of the air–TBC interface was performed with a simulated probe tip measurement. First, the TBC interface area for each sample was extracted and segmented with the IsoData-Algorithm. Then, small particles (r≤3μm) were removed from the images. This was done to avoid a false surface detection in the following step. The resulting image was imported into MATLAB to calculate the surface height profile z(x). To mimic real surface roughness measurements with a scanning probe, for each lateral position *x*, the height $z_{raw}\left(x\right)$ of the first material pixel coming from the exterior towards the TBC-layer was registered. The width of the virtual tip was set to 5μm, which is comparable to real measurement probes for the observed roughness range [26]. To get the final surface profile z(x), the constant offset is subtracted

(17) $$z\left(x\right)=z_{raw}\left(x\right)-{\bar{z}}_{raw}$$

with ${\bar{z}}_{raw}$ being the average of the surface profile. From this, the root mean square (RMS) surface roughness $\sigma_{TBC}$ is calculated.

The results for the surface roughness calculation are listed in Table 1. As uncertainty of the measurement, the standard deviation of the four measurements of ±1.6μm is used. The roughness of the TBC-BC interface was not calculated in this study since this parameter is not relevant in the roughness correction presented in Section 2.4.

Since the same manufacturing technique was employed for the different coating thickness steps, the surface roughness of the sample areas should also be comparable. To reduce the possible statistical spread of the SEM measurement, the average roughness of 16.2μm is used in the roughness correction calculation for the measurement data in Section 3.6.

### 3.3. COMSOL Simulation

**Figure 8:**
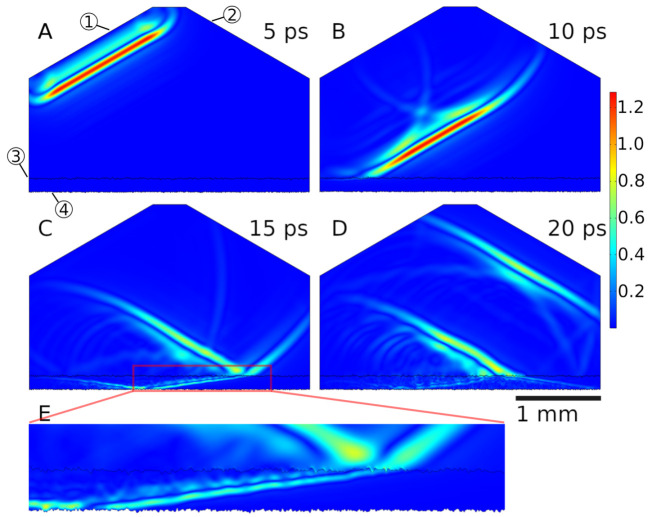
COMSOL Simulation Setup. (**A**–**D**) Snapshots of the absolute of E-Field at 5 ps increments for a simulation with rough interfaces. (**E**) shows the detail of the pulse propagation inside the coating layer at 15 ps. Balloons show sending element (1), receiving element (2), TBC interface (3) and BC interface (4). Several reflections of the pulse are visible as well as the refraction at the air–TBC interface.

To simulate THz-TDS, the experimental setup was recreated in COMSOL using the transient electromagnetic waves (ewt) interface with a 2D model. The geometry is shown in Figure 8. The wave is excited at the left angled boundary ($30{}^{\circ }$) in the form of a prescribed electric field with both in-plane (parallel) and out-of-plane (perpendicular) polarization. The lenses focusing the beam in the experimental setup are not simulated. Instead, a plane wavefront with the approximate width of the focus spot of the experimental setup was used. The pulse shape is extracted from the first reflection of the thickest sample in the real measurement data. The extracted pulse is linearly windowed to zero at the edges to avoid discontinuities. The shape of the simulation was chosen to minimize the geometric size (simulation time) by ensuring that the center of sender and receiver point to the middle of the TBC interface, thereby maximizing the illumination of the interface. Two geometric domains are present: air on top and the TBC material below. Porosities in the coating were not modeled; instead, a bulk material with an averaged refractive index calculated in Section 3.2 was used. This is essentially a simple effective medium approach. Rigorous effective medium models have been previously applied to YSZ coatings in [27]. Since the proposed method only uses the real part of the refractive index, the modeled material parameters are real-valued. This means that absorption effects are not simulated. Furthermore, the modeled material parameters are not frequency-dependent. This is a reasonable simplification, since the real part of the refractive index of YSZ is fairly constant in the investigated frequency range [27]. The outer boundaries are set as high absorption scattering with two exceptions: the sender, which has a scattering boundary condition without absorption, and the TBC–BC interface, which is set to perfect electric conductor. The width of the THz pulse is approx. 2.5 mm, which translates to an illumination projection size of about 2.9 mm. For meshing, a free triangular mesh with minimum element distance of $\frac{1}{10}$ the minimal relevant wavelength ($f_{max}=1\;\mathrm{THz}$) in the respective domain was chosen.

In this study, two types of simulations were executed. The first simulated the measurement with flat interfaces for both TBC and BC. Here, four different thicknesses were considered, equal to the SEM measurement of the thickness in Section 3.2 and refractive indices according to Appendix A.5. These simulations were used to verify the angle-correction method. The results are discussed in Section 3.4. The second type of simulation used rough interfaces for TBC and BC with varying roughness to verify the correction presented in Section 2.4. Here, the thickness and refractive index is kept constant (parameters as in flat simulation for “7 mils”). The rough interface was generated by adapting an algorithm presented in [28], allowing the creation of random rough curves with specified RMS roughness σ. Table 2 shows an overview of the simulations and used parameters.

**Figure 9:**
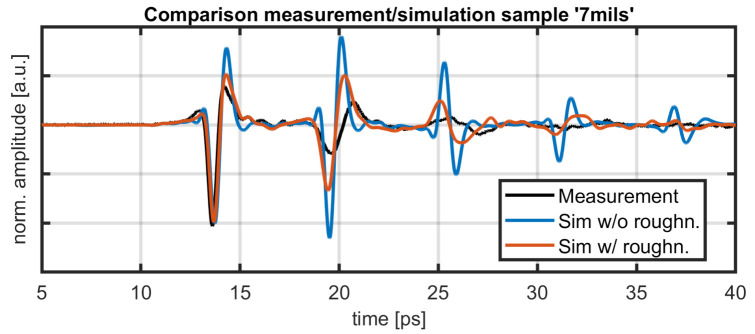
Comparison between simulation and measurement. Shown are measurement data (black), simulated data “sim. 7 mils” (blue) and simulated data “rough3” (red). The amplitude was normalized at the negative peak of the first reflection.

A comparison between real measurement data and simulated data of the same sample is shown in Figure 9. The distances between reflections show good agreement for both simulations compared to the measurement data. Pulse amplitudes show some discrepancies, especially for the simulation without roughness. Here, lacking losses from absorption or interface roughness, the reflection at the second interface shows a higher amplitude than the reflection at the first interface. This is consistent with expectations, since the second interface allows no transmission. The simulation with roughness shows reduced amplitudes of consecutive reflections, stemming from the diversion of parts of the beam energy away from the receiving element. Here, a slight pulse widening is also visible, caused by stronger scattering for higher frequency components. The amplitudes for the measurement data show a stronger decay with only three reflections visible. This is mainly caused by the absorption in the coating, which was not simulated in this work. For the experimental data, the pulse widening is also stronger, which is a result of a strong frequency dependence of the imaginary part of the refractive index in the THz range for YSZ [25].

### 3.4. Simulation: No Roughness

**Figure 10:**
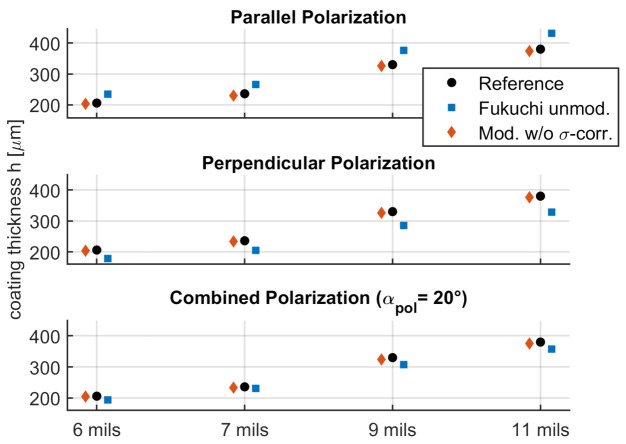
Thickness calculation for simulations without surface roughness for different polarization states. Shown are the simulated reference (black), the unmodified method by Fukuchi et al. (blue) and the adaptations proposed in this paper (red).

The results of the COMSOL model without roughness are shown in Figure 10 and in Table 3 for different states of polarization. The thicknesses are calculated without angle corrections for the refractive index calculation (but with correction for ToF) and with angle corrections for refractive index and ToF for three polarization states: parallel, perpendicular and linear polarization with a rotation of $20{}^{\circ }$ relative to perpendicular polarization (see Section 2.2). The latter signal was constructed from both the parallel and perpendicular data through superposition.

For all polarization states and thicknesses, the modified method shows good agreement with the reference values. The average error is 1.3%, compared to 10.6% for the unmodified method.

### 3.5. Simulation: With Roughness

**Figure 11:**
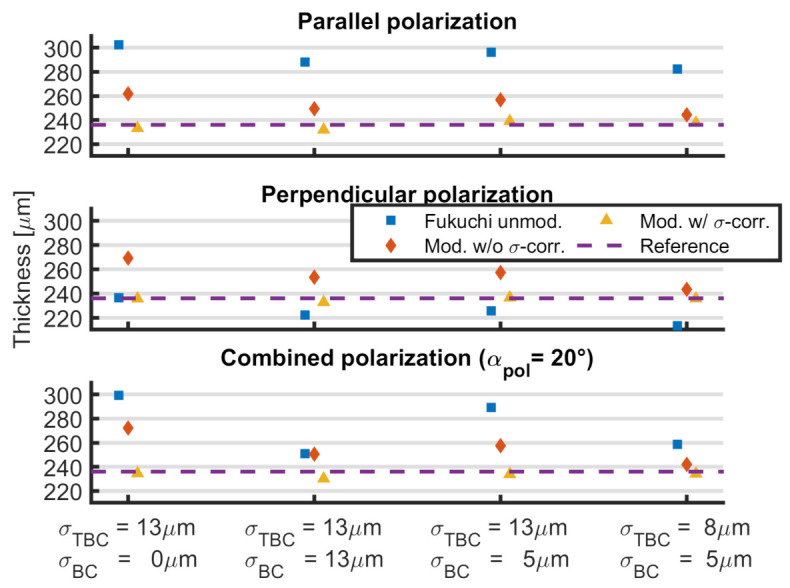
Thickness calculation for simulations with surface roughness for different polarization states. Shown are the simulated reference (purple line), the unmodified method by Fukuchi et al. (blue) and the adaptations proposed in this paper without roughness correction (red) and with roughness correction (yellow). Combined polarization angle $\alpha_{pol}=20{}^{\circ }$.

The results for the four COMSOL simulations with rough interfaces are shown in Figure 11 and Table 4. Here, the unmodified method by Fukuchi et al. shows an average error of 15%. The highest deviation is observed for the case of parallel polarization and the lowest for perpendicular polarization. For the combined polarization case, the results depend on the difference between the roughnesses of the two interfaces.

The modified method without roughness correction shows an average error of 8%. As in the case for the unmodified method, the error is large for differing interface roughnesses.

The results for the modified algorithm with roughness correction show an error of under 1% on average with a maximum absolute error of −6μm. This shows the importance of the consideration of surface roughness in THz-TDS measurements.

**Figure 12:**
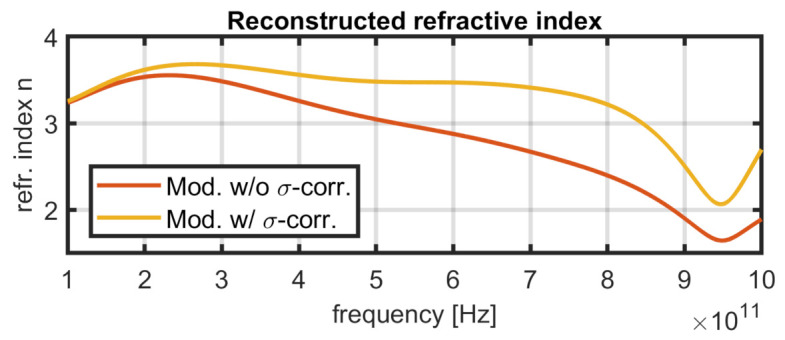
Influence of the roughness correction. Data shown are from simulation “rough3”. The simulated refractive index is approx. n=3.7.

The influence of the correction on the calculated refractive index is plotted in Figure 12. Without correction, the roughness effects lead to increasing deviations in the calculated refractive index for higher frequencies and therefore smaller wavelengths. The roughness correction factor increases with the frequency and keeps the refractive index almost constant for a wider range.

### 3.6. Measurement Data

**Figure 13:**
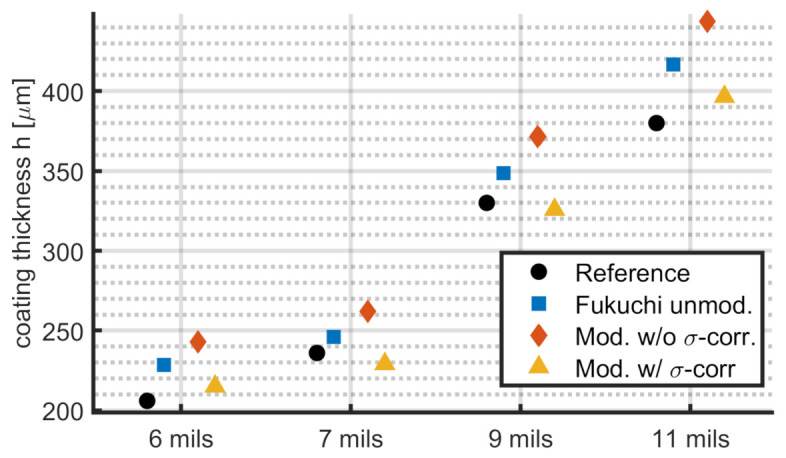
Thickness calculation for experimental data. Shown are the SEM reference (black), the unmodified method by Fukuchi et al. (blue) and the adaptations proposed in this paper without roughness correction (red) and with roughness correction (yellow).

The results for the layer thickness calculation of the sample data are shown in Figure 13 and Table 5. Using the values from the SEM scans as reference, the average error of the unmodified method is 7.6% compared to 14.5% for the modified method without roughness correction and 3.3% for the modified method with roughness correction. The unmodified algorithm shows a lower error than in the simulated data, and the modified algorithm without roughness correction has the biggest deviation. The simulations that demonstrate this behavior best are “rough2” and “rough3” for the case for perpendicular and for combined polarization. Since the surface roughness of the sample is higher compared to the simulation with roughness, a bigger error for the methods lacking roughness corrections is plausible.

For all sample measurements, the results for the modified algorithm including roughness corrections are closest to the reference SEM measurements. The absolute error ranges from 4μm to 17μm. The relative error ranges from 1.2% to 4.5%.

## 4. Discussion

In this paper, we presented a nondestructive method to determine the thickness of dielectric coatings using THz-TDS in reflection mode at non-perpendicular incidence. The method was verified with simulations and with experimental measurement data and shows good agreement with reference measurements. If the sample surface is flat in relation to the wavelength range used, the method only uses the THZ-TDS time signal as input, together with the known angle of incidence and polarization angle. In cases where the surface roughness of the sample cannot be neglected, we derived a correction term for the method requiring the RMS roughness of the surface’s additional input parameter. In both cases neither the porosity of the coating nor the refractive index needs to be known. The measurement method is not limited to TBCs and can be used for all dielectric layers with sufficient thickness to allow for separation of the multiple reflections.

Computationally, the method is fast enough to enable real-time and on-line measurement resulting in a high potential for process automation.

Several further research steps present themselves. The validation of the method for a range of sample coatings and material combinations can be used to explore the limits of the proposed algorithm. Simulation and analysis of the influence of focusing the THz beam—as is the case in the experimental setup—could improve the results. A thorough investigation of the influence of polarization state in THz-TDS measurements could lead to improvements in THz-TDS experimental setups. The combination of ellipsometric techniques with THz-TDS has the potential to extract further parameters from measurement data and thereby increase the applications. A roughness measurement with THz-TDS alone could be accomplished with a setup that allows a range of different angles of incidence by comparing the measured spectrum with reference data of surfaces with known roughness.

## Figures and Tables

**Table 1:** Results of SEM analysis: thickness of coating *h*, measured porosity ϕ, real part of refractive index $n_{real}$ and measured surface roughness $\sigma_{TBC}$.

Sample	thickn. *h* [μm]	meas. por. ϕ [%]	refr. ind. $n_{\mathrm{real}}$	surf. roughn. $\sigma_{\mathrm{TBC}}$ [μm]
6 mils	206±10	16.5±1.6	3.874±0.466	18.4±1.6
7 mils	236±10	19.0±1.3	3.872±0.412	12.4±1.6
9 mils	330±10	21.7±2.8	3.871±0.296	19.9±1.6
11 mils	380±10	18.3±1.4	3.870±0.259	14.1±1.6

**Table 2:** Performed simulations and used parameters: coating thickness $h_{TBC}$, real part of refractive index $n_{real}$, RMS roughness of TBC interface $\sigma_{TBC}$ and RMS roughness of BC interface $\sigma_{BC}$.

Sample	Name	$h_{\mathrm{TBC}}$ [μm]	$n_{\mathrm{real}}$	$\sigma_{\mathrm{TBC}}$ [μm]	$\sigma_{\mathrm{BC}}$ [μm]
flat	sim. 6 mils	206	3.64	-	-
	sim. 7 mils	236	3.71	-	-
	sim. 9 mils	330	3.75	-	-
	sim. 11 mils	380	3.81	-	-
rough	rough1	236	3.71	13	0
	rough2	236	3.71	13	13
	rough3	236	3.71	13	5
	rough4	236	3.71	8	5

**Table 3:** Thickness calculation for simulations without surface roughness for different polarization states. Comparison between modeled (real) thickness ($h_{ref}$) and errors for reconstructed thickness with the unmodified method by Fukuchi et al. ($\Delta h_{o}$) and the modified version ($\Delta h_{m}$) for inclined incidence. All values are in μm.

		Parallel		Perpendicular		Combined	
**sim. Sample**	$h_{\mathrm{ref}}$	$\Delta h_{o}$	$\Delta h_{m}$	$\Delta h_{o}$	$\Delta h_{m}$	$\Delta h_{o}$	$\Delta h_{m}$
6 mils	206	+29	−2	−27	−2	−12	−2
7 mils	236	+30	−5	−31	−2	−5	−3
9 mils	330	+46	−4	−45	−4	−22	−6
11 mils	380	+51	−7	−51	−4	−23	−4

**Table 4:** Thickness calculation for simulations with surface roughness for different polarization states of the incident pulse. The layer thickness is 236μm for all models. The errors are tabulated for the original method by Fukuchi et al. ($\Delta h_{o}$), the modified version without roughness correction ($\Delta h_{m}$) and the modified version with roughness correction ($\Delta d_{m,r}$). All values are in μm.

	Method		rough1	rough2	rough3	rough4
		$\sigma_{\mathrm{TBC}}$	**13**	**13**	**13**	**8**
		$\sigma_{\mathrm{BC}}$	**0**	**13**	**5**	**5**
	$\Delta h_{o}$		+66	+52	+60	+46
par.	$\Delta h_{m}$		+26	+13	+21	+8
	$\Delta d_{m,r}$		−3	−4	+3	+2
	$\Delta h_{o}$		+0	−14	−11	−23
perp.	$\Delta h_{m}$		+33	+17	+21	+8
	$\Delta d_{m,r}$		+0	−3	+1	+0
	$\Delta h_{o}$		+63	+15	+53	+23
comb.	$\Delta h_{m}$		+36	+15	+22	+6
	$\Delta d_{m,r}$		−1	−6	−2	−2

**Table 5:** Results for measurement data of the four sample areas. The reference measurements are tabulated from SEM data ($h_{ref}$) and errors for the original method by Fukuchi et al. ($\Delta h_{o}$), the modified version without roughness correction ($\Delta h_{m}$) and the modified version with roughness correction ($\Delta d_{m,r}$). All values are in μm.

Sample	$h_{\mathrm{ref}}$	$\Delta h_{o}$	$\Delta h_{m}$	$\Delta d_{m,r}$
6 mils	206	+22	+36	+9
7 mils	236	+10	+26	−7
9 mils	330	+19	+42	−4
11 mils	380	+37	+64	+17

**Figure A1:**
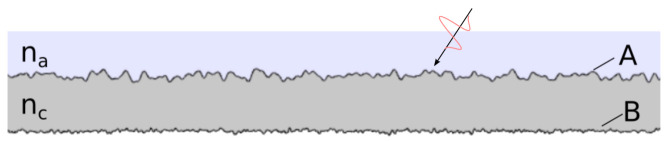
Naming scheme for calculation of rough layer reflections.

## Data Availability

The data supporting the findings of this paper are available from the authors at request.

## Abbreviations

BC	Bond coat
NDT	Nondestructive testing
RMS	Root mean square
SEM	Scanning electron microscopy
TBC	Thermal barrier coating
TDS	Time-domain spectroscopy
THz	Terahertz
THz-TDS	Terahertz time-domain spectroscopy
ToF	Time-of-flight
YSZ	Yttria-stabilized zirconia

## Appendix A. Detailed Calculations

### Appendix A.1. Angle in Coating

For the following calculation, a cosine relation between $\theta_{a}$ and $\theta_{c}$ is useful. From Equation (7) and trigonometric relation follows

(A1) $$\cos\theta_{c}=\sqrt{1-\sin{\theta_{c}}^{2}}=\sqrt{1-{\left(\frac{n_{a}}{n_{c}}\right)}^{2}\;\left(1-\cos{\theta_{a}}^{2}\right)}.$$

### Appendix A.2. Angled Incidence with Perpendicular Polarization

The Fresnel coefficients for perpendicular polarization are

(A2) $$\begin{array}{ccc} r_{⊥,ac}=\frac{n_{a}\;\cos\theta_{a}-n_{c}\;\cos\theta_{c}}{n_{a}\;\cos\theta_{a}+n_{c}\;\cos\theta_{c}}\; & & r_{⊥,ca}=\frac{n_{c}\;\cos\theta_{c}-n_{a}\;\cos\theta_{a}}{n_{c}\;\cos\theta_{c}+n_{a}\;\cos\theta_{a}}\\ t_{⊥,ac}=\frac{2\;n_{a}\;\cos\theta_{a}}{n_{a}\;\cos\theta_{a}+n_{c}\;\cos\theta_{c}}\; & & t_{⊥,ca}=\frac{2\;n_{c}\;\cos\theta_{c}}{n_{c}\;\cos\theta_{c}+n_{a}\;\cos\theta_{a}}. \end{array}$$

Γ of Equation (2) transforms with Equation (A2) in case of perpendicular polarization to

(A3) $$\begin{array}{cc} \; & \Gamma_{⊥}=-\frac{r_{⊥,ac}\;r_{⊥,ca}}{t_{⊥,ac}\;t_{⊥,ca}}=\frac{{\left(n_{a}\;\cos\theta_{a}-n_{c}\;\cos\theta_{c}\right)}^{2}}{4\;n_{a}\;n_{c}\;\cos\theta_{a}\;\cos\theta_{c}}\\ \; & =\frac{{\left(n_{a}\;\cos\theta_{a}-n_{c}\;\sqrt{1-{\left(\frac{n_{a}}{n_{c}}\right)}^{2}\;\left(1-\cos{\theta_{a}}^{2}\right)}\right)}^{2}}{4\;n_{a}\;n_{c}\;\cos\theta_{a}\;\sqrt{1-{\left(\frac{n_{a}}{n_{c}}\right)}^{2}\;\left(1-\cos{\theta_{a}}^{2}\right)}}. \end{array}$$

Further simplification of Equation (A3) leads, together with $k_{a}=\cos\theta_{a}$, to -4.6cm0cm

(A4) $$\begin{array}{cc} \Rightarrow \; & \Gamma_{⊥}\;4\;n_{a}\;n_{c}\;k_{a}\;\sqrt{1-{\left(\frac{n_{a}}{n_{c}}\right)}^{2}\;\left(1-k_{a}^{2}\right)}={\left(n_{a}\;k_{a}-n_{c}\;\sqrt{1-{\left(\frac{n_{a}}{n_{c}}\right)}^{2}\;\left(1-k_{a}^{2}\right)}\right)}^{2}\\ \Leftrightarrow \; & \left(2\;\Gamma_{⊥}+1\right)\;2\;n_{a}\;n_{c}\;k_{a}\;\sqrt{\cdots }=n_{c}^{2}-n_{a}^{2}+2\;n_{a}^{2}\;k_{a}^{2}\\ \Leftrightarrow \; & n_{c}^{4}+\underset{A_{⊥}}{\underset{︸}{2\;n_{a}^{2}\;\left(-1-8\;k_{a}^{2}\;\Gamma_{⊥}\;\left(\Gamma_{⊥}+1\right)\right)}}\;n_{c}^{2}+\underset{B_{⊥}}{\underset{︸}{n_{a}^{4}\;\left(1+16\;k_{a}^{2}\;\Gamma_{⊥}\left(1+\Gamma_{⊥}^{2}-k_{a}^{2}\;\Gamma_{⊥}^{2}-k_{a}^{2}\right)\right)}}=0. \end{array}$$

The substitution of $x=n_{c}^{2}>1$ reduces Equation (A4) to a quadratic equation. Solving and resubstitution *x* leaves only one physically meaningful solution for $A_{⊥}<0$ and $B_{⊥}>0$:

(A5) $$\begin{array}{cc} n_{⊥,c} & =+\sqrt{-\frac{A_{⊥}}{2}+\sqrt{\frac{A_{⊥}^{2}}{4}-B_{⊥}}}\\ \; & =n_{a}\sqrt{1+8\;k_{a}^{2}\;\Gamma \left(\Gamma +1\right)+k_{a}^{2}\;\sqrt{16\;\Gamma \;\left(4\;\Gamma^{3}+9\;\Gamma^{2}+4\;\Gamma +1\right)}}. \end{array}$$

### Appendix A.3. Angled Incidence with Parallel Polarization

The Fresnel coefficients for perpendicular polarization are

(A6) $$\begin{array}{ccc} r_{\|,ac}=\frac{n_{c}\;\cos\theta_{a}-n_{a}\;\cos\theta_{c}}{n_{c}\;\cos\theta_{a}+n_{a}\;\cos\theta_{c}}\; & & r_{\|,ca}=\frac{n_{a}\;\cos\theta_{c}-n_{c}\;\cos\theta_{a}}{n_{a}\;\cos\theta_{c}+n_{c}\;\cos\theta_{a}}\\ t_{\|,ac}=\frac{2\;n_{a}\;\cos\theta_{a}}{n_{c}\;\cos\theta_{a}+n_{a}\;\cos\theta_{c}}\; & & t_{\|,ca}=\frac{2\;n_{c}\;\cos\theta_{c}}{n_{a}\;\cos\theta_{c}+n_{c}\;\cos\theta_{a}}. \end{array}$$

Γ from Equation (2) becomes with Equation (A6) in case of parallel polarization

(A7) $$\begin{array}{cc} \Gamma_{\|} & =-\frac{r_{\|,ac}\;r_{\|,ca}}{t_{\|,ac}\;t_{\|,ca}}=\frac{{\left(n_{c}\cos\theta_{a}-n_{a}\cos\theta_{c}\right)}^{2}}{4\;n_{a}\;n_{c}\;\cos\theta_{a}\;\cos\theta_{c}}\\ \; & =\frac{{\left(n_{c}\;\cos\theta_{a}-n_{a}\;\sqrt{1-{\left(\frac{n_{a}}{n_{c}}\right)}^{2}\;\left(1-\cos{\theta_{a}}^{2}\right)}\right)}^{2}}{4\;n_{a}\;n_{c}\;\cos\theta_{a}\;\sqrt{1-{\left(\frac{n_{a}}{n_{c}}\right)}^{2}\;\left(1-\cos{\theta_{a}}^{2}\right)}} \end{array}$$

This leads to an equation of eighth order in $n_{c}$ and should be solved numerically since the high number of cases prohibits the formulation of a closed solution.

As an alternative, the following approximation can be made. Beginning from Equation (A6) with the substitutions $n^{*}=\frac{n_{a}}{n_{c}}$ and $k_{a}=\cos\theta_{a}$, one gets

(A8) $$\Gamma_{\|}=\frac{{\left(k_{a}-n^{*}\;\sqrt{1-n^{*2}\;\left(1-k_{a}^{2}\right)}\right)}^{2}}{4\;n^{*}\;k_{a}\;\sqrt{1-n^{*2}\;\left(1-k_{a}^{2}\right)}}.$$

For an assumed ratio of refractive indices $n^{*}=\frac{1}{4}$ (This is equivalent to a speed of propagation of $\frac{1}{4}\;c_{vac}$ in the coating and a good estimation for the YSZ coating with porosities) and for the angle of incidence $k_{a}=\cos45=0.707$, the root in Equation (A8) becomes

(A9) $$\sqrt{1-n^{*2}\;\left(1-k_{a}^{2}\right)}=0.984\approx 1$$

and can thus be omitted in most practical cases. With this assumption Equation (A8) leads to

(A10) $$\Gamma_{\|}=\frac{{\left(k_{a}-n^{*}\right)}^{2}}{4\;n^{*}\;k_{a}}.$$

Furthermore, with further calculations

(A11) $$n_{\|,c}=\frac{n_{a}}{n^{*}}=\frac{n_{a}}{k_{a}\left(2\;\Gamma_{\|}+1-2\;\sqrt{\Gamma_{\|}^{2}+\Gamma_{\|}}\right)}.$$

### Appendix A.4. Roughness Correction for Inclined Incidence

Under the assumption of an infinite surface and a gentle roughness curve (tangent plane approach), the spectrum of a reflection at a rough interface $F^{R}$ can be described [21] by averaging the phase shift $\delta \phi_{r}$ caused by the surface profile

(A12) $$F^{R}=F^{S}\;\left|\langle \exp\left(i\delta \phi_{r}\right)\rangle \right|$$

with $F^{S}$ being the spectrum for a completely flat surface. Using the Rayleigh roughness parameter $Ra_{r}$, the correction term can be expressed by

(A13) $$\left|\langle \exp\left(i\delta \phi_{r}\right)\rangle \right|=\exp\left(-\frac{Ra_{r}^{2}}{2}\right).$$

The Rayleigh roughness parameter itself can be calculated from the wave number $k=\frac{2\pi }{\lambda }=2\pi \;\frac{f}{c_{vac}}$, the RMS roughness σ and the angle of incidence $\theta_{a}$

(A14) $$Ra_{r}=k\;\sigma \;\cos\theta_{a}.$$

Since the calculation method presented in this study is not only dependent on a simple interface reflection but on multiple reflections at different interfaces including transmission in a layer with a high refractive index (n≈4), the roughness correction has to be extended to this case. Pinel et al. [21] presented a calculation for the case of two stacked, uncorrelated rough surfaces A and B (see Figure A1), which fits well for the case of a rough TBC layer ($n_{c}$) with air ($n_{a}$) on top. The incidence of the pulse is from the top.

The three pulse spectra $F_{1-3}^{R}$ have to be adjusted individually. For the direct reflection $F_{1}$, the Rayleigh parameter is simply (compare Equation (A14))

(A15) $$Ra_{r,1}=k_{a}\;\sigma_{A}\;\cos\theta_{a}$$

with $k_{a}=n_{a}\;k_{0}=n_{a}\;\frac{2\pi \;f}{c_{vac}}$ as the wave number in air, $\sigma_{A}$ the RMS roughness of interface A and $\theta_{a}$ as the angle of incidence relative to the (smooth) surface normal.

The corrections for the spectra of the multiple reflections $F_{m=2}$ and $F_{m=3}$ have to consider the transmission and roughness of interface B. The Rayleigh parameter becomes

(A16) $$Ra_{r,m}=\sqrt{2{\left(Ra_{t,\pm A}\right)}^{2}+\left(m-1\right){\left(Ra_{r,+B}\right)}^{2}+\left(m-2\right){\left(Ra_{r,-A}\right)}^{2}}.$$

Here, the total Rayleigh parameters of the pulses are determined by several Rayleigh parameters of the interfaces in the path of travel. The transmission through interface A is considered by

(A17) $$Ra_{t,\pm A}=k_{0}\;\sigma_{A}\;\frac{\left|n_{a}\;\cos\theta_{a}-n_{c}\;\cos\theta_{c}\right|}{2}$$

with the angle inside the layer $\theta_{c}$, $n_{a}$ the index of refraction above interface A and $n_{c}$ the index of refraction between interfaces A and B.

The reflection at interface B is accounted by

(A18) $$Ra_{r,+B}=k_{c}\;\sigma_{B}\;\cos\theta_{c}$$

with the roughness of the surface B $\sigma_{B}$ and the wave number in the coating $k_{c}=n_{c}\;k_{0}$.

Finally, the reflection at the underside of interface A has the Rayleigh parameter

(A19) $$Ra_{r,-A}=k_{c}\;\sigma_{A}\;\cos\theta_{c}.$$

This results in a Rayleigh parameter for $F_{2}^{R}$ of

(A20) $$Ra_{r,2}=\sqrt{2{\left(Ra_{t,\pm A}\right)}^{2}+{\left(Ra_{r,+B}\right)}^{2}}$$

and for $F_{3}^{R}$ of

(A21) $$Ra_{r,3}=\sqrt{2{\left(Ra_{t,\pm A}\right)}^{2}+2{\left(Ra_{r,+B}\right)}^{2}+{\left(Ra_{r,-A}\right)}^{2}}.$$

With this, the factor Γ is corrected for roughness: The measured value is

(A22) $$\Gamma^{R}=\frac{F_{1}^{R}\;F_{3}^{R}}{{\left(F_{2}^{R}\right)}^{2}}.$$

Using Equation (A12) gives

(A23) $$\Gamma^{R}=\Gamma^{S}\;\underset{K_{R}}{\underset{︸}{\exp\left(-\frac{1}{2}\left({\left(Ra_{r,1}\right)}^{2}+{\left(Ra_{r,3}\right)}^{2}-2{\left(Ra_{r,2}\right)}^{2}\right)\right)}}$$

with $\Gamma^{S}$ as the parameter at the interface without roughness and $K_{R}$ as the correction factor. Simplifying the exponential term in Equation (A23) gives

(A24) $$K_{R}=\exp\left(-\frac{1}{2}\left({\left(Ra_{r,1}\right)}^{2}-{\left(Ra_{t,\pm A}\right)}^{2}+3{\left(Ra_{r,-A}\right)}^{2}\right)\right).$$

For the remaining unknown parameter of the refractive index of the coating, Section 2.4 describes an iterative method of estimation. There, $\sigma_{A}$ is renamed as $\sigma_{TBC}$ for clarity.

### Appendix A.5. Determination of n c from ToF and Known Thickness

The thickness of the TBC layer $h_{c}$ is a function of the ToF between two consecutive pulses Δt with

(A25) $$h_{c}=\left(\frac{c_{vac}}{n_{c}}\;\frac{\Delta t}{2}\right)\;\cos\theta_{c}.$$

Insertion of Equation (A1) in Equation (A25) leads to a relation of fourth order in $n_{c}$. Solving results in one physically meaningful solution for $n_{c}$:

(A26) $$n_{c}=+\sqrt{\frac{a}{2}+\sqrt{\frac{a^{2}}{4}-b}}$$

with $a={\left(\frac{c_{vac}\;\Delta t}{2\;h_{c}}\right)}^{2}$ and $b={\left(\frac{c_{vac}\;\Delta t\;n_{a}}{2\;h_{c}}\right)}^{2}\;\left(1-\cos{\theta_{a}}^{2}\right)$.
